# Construction of Mutant Glucose Oxidases with Increased Dye-Mediated Dehydrogenase Activity

**DOI:** 10.3390/ijms131114149

**Published:** 2012-11-02

**Authors:** Yohei Horaguchi, Shoko Saito, Katsuhiro Kojima, Wakako Tsugawa, Stefano Ferri, Koji Sode

**Affiliations:** 1 Department of Biotechnology, Graduate School of Engineering, Tokyo University of Agriculture and Technology, 2-24-16 Nakamachi, Koganei, Tokyo 184-8588, Japan; E-Mails: 50011641137@st.tuat.ac.jp (Y.H.); 50012641118@st.tuat.ac.jp (S.S.); tsugawa@cc.tuat.ac.jp (W.T.); stefano@cc.tuat.ac.jp (S.F.); 2 Ultizyme International Ltd., 1-13-16, Minami, Meguro, Tokyo 152-0013, Japan; E-Mail: kojima@ultizyme.jp

**Keywords:** glucose oxidase, glucose dehydrogenase, FAD, oxygen, site directed mutagenesis

## Abstract

Mutagenesis studies on glucose oxidases (GOxs) were conducted to construct GOxs with reduced oxidase activity and increased dehydrogenase activity. We focused on two representative GOxs, of which crystal structures have already been reported—*Penicillium amagasakiense* GOx (PDB ID; 1gpe) and *Aspergillus niger* GOx (PDB ID; 1cf3). We constructed oxygen-interacting structural models for GOxs, and predicted the residues responsible for oxidative half reaction with oxygen on the basis of the crystal structure of cholesterol oxidase as well as on the fact that both enzymes are members of the glucose/methanol/choline (GMC) oxidoreductase family. Rational amino acid substitution resulted in the construction of an engineered GOx with drastically decreased oxidase activity and increased dehydrogenase activity, which was higher than that of the wild-type enzyme. As a result, the dehydrogenase/oxidase ratio of the engineered enzyme was more than 11-fold greater than that of the wild-type enzyme. These results indicate that alteration of the dehydrogenase/oxidase activity ratio of GOxs is possible by introducing a mutation into the putative functional residues responsible for oxidative half reaction with oxygen of these enzymes, resulting in a further increased dehydrogenase activity. This is the first study reporting the alteration of GOx electron acceptor preference from oxygen to an artificial electron acceptor.

## 1. Introduction

Glucose oxidase (β-d-glucose:oxygen 1-oxidoreductases, E.C. 1.1.3.4; GOx) is a flavoprotein that catalyzes the oxidation of β-d-glucose at its first hydroxyl group by using molecular oxygen as the electron acceptor to produce glucono-β-lactone and hydrogen peroxide. GOx is a homodimeric enzyme, with a flavin adenine dinucleotide (FAD) molecule noncovalently, but tightly, bound at the active site of each subunit. GOx is a representative enzyme of the glucose/methanol/choline (GMC) oxidoreductase family, which shares a homologous structural backbone, including an ADP-binding βαβ-fold close to the amino terminus and five other segments of a conserved sequence dispersed throughout the primary sequence. This large and diverse family includes many industrially important enzymes, especially in the field of diagnostics, such as cholesterol oxidase (ChOx), alcohol oxidase, amino acid oxidase, and pyranose oxidase. GOx was originally isolated and produced from fungi. The natural production of GOxs by fungi, usually by *Aspergillus niger*[1] or *Penicillium* spp. [2], produces an extracellular enzyme that is over 10% glycosylated. Recombinant production of GOxs in yeast, such as *Saccharomyces cerevisiae*[3] and *Hansenula polymorpha*[4], often results in hyperglycosylation, with a concomitant decrease in enzymatic activity. Recombinant production of *P. amagasakiense*-derived GOx in *Escherichia coli* has been reported [5], but it has resulted in inactive GOx in the inclusion bodies, which can be solubilized and refolded *in vitro* in low yield to an active unglycosylated enzyme with properties very similar to the native enzyme.

Since the report on the first enzyme sensor [6] employing GOx and an oxygen electrode for glucose monitoring, extensive studies have been performed to develop improved enzyme-based systems for monitoring glycemic levels, along with the employment of various enzymes catalyzing glucose oxidation [7]. The first-generation blood glucose monitoring systems employed oxygen as the electron acceptor, and glucose concentration was determined by monitoring either the consumption of oxygen or liberation of hydrogen peroxide. The second-generation sensors employed artificial electron acceptors (also referred to as electron mediators or redox dyes) instead of oxygen to avoid interference from other redox species. However, electron mediator-type enzyme sensors employing oxidases are inherently influenced by the amount of oxygen dissolved in the sample. The high reactivity of oxidases with oxygen limits their potential applications for biosensors employing artificial electron acceptors. Therefore, oxidases that are relatively less oxygen-sensitive would be greatly advantageous for the development of amperometric enzyme sensors.

We have previously reported on the engineering of fructosyl amino acid oxidase [8] (FAOD) and fructosyl peptide oxidase [9] (FPOX). These enzymes are used in the biosensing of glycated proteins for the diagnosis and assessment of treatment response in diabetes. To engineer FAOD and FPOX, we introduced mutations in amino acid residues that constitute up the proton relay system, which is responsible for the transfer of electrons to oxygen. We have also described the construction of engineered FPOX, which showed drastically decreased oxidase activity as well as increased dehydrogenase activity.

This successful strategy inspired us to engineer GOxs. In this study, we conducted mutagenesis analyses of GOxs in order to construct GOxs with reduced oxidase activity, which is the oxidative half reaction using oxygen as the electron acceptor, and increased dehydrogenase activity, which is the oxidative half reaction using artificial electron acceptor. We focused on two representative GOxs, of which crystal structures have been reported—*P. amagasakiense*-derived GOx (PDB ID; 1gpe) and *A. niger*-derived GOx (PDB ID; 1cf3). By constructing oxygen-interacting structural model of GOxs, we predicted the residues responsible for oxydative half reaction with oxygen, on the basis of the crystal structure of ChOx with an oxygen molecule as well as on the fact that both enzymes are members of the GMC oxidoreductase family. Rational amino acid substitution resulted in the construction of an engineered GOx with drastically decreased oxidase activity and increased dehydrogenase activity, which was higher than that of the wild-type enzyme. As a result, the dehydrogenase/oxidase ratio of the engineered enzyme was more than 11-fold greater than that of the wild-type enzyme.

## 2. Results and Discussion

### 2.1. Oxygen-Interacting Structural Models for GOxs

**Figure 1:**
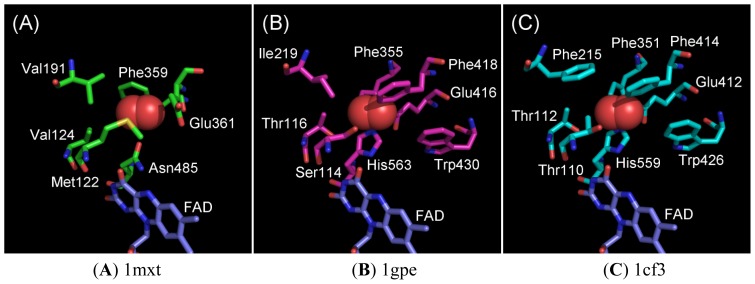
3D structure of cholesterol oxidase from *Streptomyces* sp. (PDB ID: 1mxt) and oxygen- interacting structural models for glucose oxidases. The oxygen molecule (red ball) of 1mxt was superimposed on 1gpe and 1cf3. These images were produced using PyMol 1.5.0.3 (Schrödinger, LLC., New York, NY, USA).

The currently available GOx crystal structures do not contain oxygen molecule; hence, we constructed oxygen-interacting structural models for GOxs based on the superimposition of ChOx (1MXT) with 2 representative GOxs—1gpe and 1cf3. The superimposition was carried out using UCSF Chimera Ver.1.3 [10] by fixing the position of FAD in their structures. Figures 1A shows an oxygen molecule in crystal structure of ChOx (PDB ID; 1mxt) [11] and Figure 1B,C show the oxygen molecule superimposed on 1gpe and 1cf3, respectively.

From the analysis of the 3D structure of ChOx, we predicted the following six residues close (within 5 Å) to the oxygen molecule: Met122, Val124, Val191, Phe359, Glu361, and Asn485. The superimposed structure of GOx revealed the following 8 residues are located close to the oxygen molecule in 1gpe: Ser114, Thr116, Ile219, Phe355, Glu416, Phe418, Trp430, and His563, and in 1cf3: Thr110, Thr112, Phe215, Phe351, Glu412, Phe414, Trp426, and His559. Among the residues observed in the GOx oxygen-interacting models, Glu416/Glu412, Phe418/Phe414, and Trp430/Trp426 are unique to GOxs, and no corresponding residues were found in ChOx. Among these eight residues, six residues are conserved between 1gpe and 1cf3 GOx. Considering the elucidated oxygen-interacting structural models for GOxs, mutagenesis analyses were performed for each of these eight residues (Ser114, Thr116, Ile219, Phe355, Glu416, Phe418, Trp430, and His563) for 1gpe, and for five residues (Thr110, Thr112, Phe351, Phe414, and Trp426) for 1cf3, which were predicted to be the putative functional residues responsible for oxidative half reaction with oxygen.

### 2.2. Ala Substitutions within the Putative Functional Residues Responsible for Oxidative Half Reaction with Oxygen for 1gpe and 1cf3

All enzyme samples used in this study, including the wild-type enzymes, were recombinantly prepared in *E. coli*. All samples were expressed as IBs and were refolded as described in the Experimental Section. The refolded enzyme samples were recovered as water-soluble proteins with single-band quality, as determined by SDS-PAGE analysis (data not shown). These samples were subjected to analyses of glucose oxidase and dehydrogenase activities. Table 1 summarizes the oxidase and dehydrogenase activities of the crude preparations of wild-type enzymes (1gpe and 1cf3), eight Ala-substituted mutants for 1gpe, and four Ala-substituted mutants for 1cf3 investigated in this study. The specific activities were determined using 100 mM glucose for 1gpe and 200 mM for 1cf3, respectively, considering their *K*_m_ values for glucose of the native enzymes (5.7 mM for 1gpe [5] and 30 mM for 1cf3 [12]).

The dehydrogenase/oxidase ratio for the wild-type enzymes was 18% for 1gpe and 27% for 1cf3. Most of the 1gpe GOx mutants showed drastic reductions in enzyme activities. Thr116Ala, Ile219Ala, Glu416Ala, and His563Ala scarcely showed either oxidase or dehydrogenase activities. Phe355Ala and Phe418Ala showed detectable enzyme activities. However, the reductions in oxidase activities were significant, with dehydrogenase/oxidase ratios of 145% and 127% for Phe355Ala and Phe418Ala GOx, respectively. Among these mutants, Ser114Ala and Trp430Ala showed relatively high dehydrogenase activities. Both mutants showed decreased oxidase activities compared with those of the wild-type enzyme (31% for Ser114Ala and 0.9% for Trp430Ala). Dehydrogenase activities for Ser114Ala and Trp430Ala were 380% and 12%, respectively, compared with that of the wild-type enzyme. Consequently, the dehydrogenase/oxidase ratios for Ser114Ala and Trp430Ala drastically increased to more than 12.3 and 13.8 fold greater than that of the wild-type, respectively.

Five Ala-substituted mutants, Thr110Ala, Thr112Ala, Phe351Ala, Phe414Ala, and Trp426Ala, were constructed for 1cf3, which corresponded to Ser114Ala, Thr116Ala, Phe355Ala, Phe418Ala, and Trp430Ala of 1gpe. Phe351Ala and Phe414Ala showed scarce oxidase activity, but they showed detectable dehydrogenase activity, and consequently, the dehydrogenase/oxidase ratios were increased. Thr110Ala, Thr112Ala and Trp426Ala showed oxidase and dehydrogenase activities. These mutants showed decreased oxidase activity (30% for Thr110Ala, 3.9% for Thr112Ala and 14% for Trp426Ala). Dehydrogenase activities for Thr110Ala, Thr112Ala and Trp426Ala were 200%, 17.5% and 87%, respectively, compared with that of the wild-type 1cf3. Consequently, the dehydrogenase/oxidase ratios for Thr110Ala, Thr112Ala and Trp426Ala drastically increased to more than 6.6, 4.4 and 6.1 fold greater than that of the wild-type, respectively.

Several GOx mutants both for 1gpe and for 1cf3 have been previously reported [13–18]. Some Ala-substituted mutants of 1gpe that were constructed and characterized in this study have also been previously reported [18]; these include Phe418Ala, Trp430Ala, and His563Ala. In these studies, Phe418Ala and Trp430Ala were reported to have a drastic decrease in oxidase activity. His563Ala was reported to lack oxidase activity. The results of oxidase activity of our study are in good agreement with those of previous reports. However, dye-mediated dehydrogenase activity had never been previously reported.

Considering that 1gpe Ser114Ala showed the highest dehydrogenase activity, further kinetic investigation was conducted using gel chromatography-purified refolded enzyme samples.

### 2.3. Characterization of Wild-Type 1gpe and Ser114Ala Mutant

**Figure 2:**
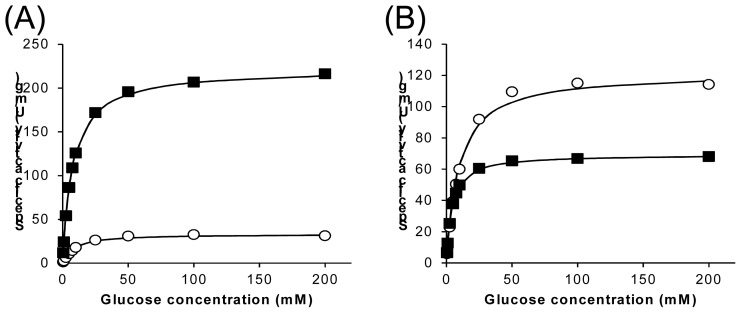
Oxidase and dehydrogenase activities of wild-type 1gpe and Ser114Ala mutant toward glucose (**A**) wild-type, (**B**) Ser114Ala mutant. Closed box: oxidase activity; open circle: dehydrogenase activity.

Figure 2A,B show the substrate-concentration dependency of oxidase and dehydrogenase activities for wild-type and the Ser114Ala mutants as purified refolded sample preparations. These figures clearly demonstrate that the Ser114Ala mutation drastically alters 1gpe enzyme properties, especially the ratio of dehydrogenase and oxidase activities. Table 2 summarizes the kinetic parameters of wild type 1gpe and Ser114Ala mutant for glucose. The wild-type enzyme sample showed higher oxidase activity (*V*_max_ = 230 U/mg) than dehydrogenase activity (33 U/mg), and the dehydrogenase/oxidase ratio was 15%. The specific activity of purified enzyme prepared form IB recovered from *E. coli* was about 20% compared with those prepared from the original fungi (925 U/mg [5]). The low activity of this preparation was probably due to low refolding efficiency. The absence of the protein glycosylation of recombinant enzyme might also contribute in the yield of inactivated enzyme during the purification procedure. The *K*_m_ values of oxidase and dehydrogenase were less than 10 mM for glucose (8.2 mM for oxidase and 9.3 mM for dehydrogenase). In contrast, Ser114Ala showed higher dehydrogenase activity than oxidase activity. Ser114Ala showed decreased oxidase activity (*V*_max_ = 70 U/mg) compared with that of the wild-type (30%). The dehydrogenase activity of Ser114Ala increased (*V*_max_ = 120 U/mg) compared with the oxidase activity; it was more than 3.6 fold greater than that of the wild-type. As a result, the dehydrogenase/oxidase ratio was approximately 170%, which is more than an 11-fold increase compared with the ratio for the wild-type enzyme (15%). The Km values of Ser114Ala for oxidase and for dehydrogenase were less than 10 mM for glucose (4.2 mM for oxidase and 9.4 mM for dehydrogenase).

These results indicate that alteration of the dehydrogenase/oxidase activity ratio of 1gpe and 1cf3 is possible by introducing mutations within the putative functional residues responsible for oxidative half reaction with oxygen of these enzymes, resulting in enzymes with further increased dehydrogenase activity. To the best of our knowledge, this is the first study reporting the alteration of GOx electron acceptor preference from an oxygen molecule to an artificial electron acceptor.

## 3. Experimental Section

### 3.1. Bacterial strains and Plasmids

*E. coli* BL21 (DE3) was used as the host strain for the expression of wild-type and mutant GOxs. The genetic information for 1gpe and 1cf3 was obtained from GenBank ID; AAD01493 and GenBank ID; ACR56326, respectively. The designed genes were obtained from GenScript (Piscataway, NJ, USA). The synthesized genes were amplified by PCR, except for the signal sequence region, by adding an *Nde*I restriction site at the amino terminus and a *Hin*dIII site downstream of the stop codon; then, the genes were inserted into the multi-cloning site of the expression vector pET-22b(+) (Merck KGaA, Darmstat, Germany).

### 3.2. Site-Directed Mutagenesis

Site-directed mutagenesis was carried out using the QuikChange Mutagenesis Kit (Agilent Technology, Santa Clara, CA, USA) in accordance with the manufacturer’s instructions. The mutations were confirmed by sequencing with an ABI Prism BigDye Terminator cycle sequencing kit v3.0 on an ABI Prism 3100 Genetic Analyzer (Applied Biosystems, Foster City, CA, USA).

### 3.3. Enzyme Preparation

Transformants with the expression vector and inserted gene encoding either the wild-type 1gpe, mutant 1gpe, wild-type 1cf3, or mutant 1cf3, without its signal sequence for secretion, were precultured in 3 mL LB medium (Ampicillin 100 μg/mL) at 37 °C aerobically for 12 h. One milliliter of the pre-culture was then inoculated into 100 mL of the same medium, and the cultures were grown aerobically in a reciprocal shaker at 37 °C. Once the OD_600_ reached 0.6 (after approximately 3 h), 0.5 mM IPTG was added for the induction, followed by 24 h cultivation at 20 °C. Cells were then centrifuged (5000× *g*, 10 min, 4 °C). Approximately 0.8 g of wet weight of cells was obtained from 100 mL culture (8 g wet cell/1 L culture). Then, these wet cells were resuspended in 3 mL of 20 mM Tris-HCl buffer (pH 8.0) with 50 mM NaCl and disrupted by ultrasonication (15 min). The sample was then centrifuged (10,000× *g* for 20 min at 4 °C), and the precipitation was collected as the insoluble fraction.

The insoluble fraction was designated as the inclusion bodies (IB), and it was subjected to the refolding procedures as described in a previous report [5].

Purified wild-type 1gpe and 1gpe Ser114Ala GOxs were prepared as follows. The refolded sample that showed GOx activity was concentrated by centrifugal ultrafiltration using Amicon Ultra-15 3K (molecular cutoff, 3 kDa). The concentrated sample was then mildly acidified by dialyzing against 20 mM sodium acetate buffer (pH 6.0) for 12 h at 4 °C; subsequently, the resultant aggregates were removed by centrifugation (17,400× *g*, 3 min 4 °C). The supernatant was again subjected to centrifugal ultrafiltration as described above, resulting in a concentrated solution. The refolded, concentrated, and acidified sample was then subjected to gel chromatography (Superdex 200 10/300 GL, with 20 mM sodium acetate buffer, pH 6.0 and 150 mM NaCl as the running buffer, at 0.4 mL/min using FPLC). All eluted fractions were recovered and dialyzed against 20 mM sodium acetate buffer (pH 6.0), and their GOx activities were determined.

### 3.4. Enzyme Assay

The glucose oxidase activities of various wild-type and mutant GOxs were investigated by determining the formation of hydrogen peroxide using 1.5 mM 4-aminoantipyrine, 1.5 mM *N*,*N*-Bis(4-sulfobutyl)-3-methylaniline disodium salt (TODB), and 2 U horseradish peroxidase/mL. The formation of the quinoneimine dye was measured at 546 nm, and 38 mM^−1^ cm^−1^ was used as the molar absorption coefficient of TODB at pH 7.0. Various concentrations of glucose were used as the substrate. The amount of an enzyme that forms 1 μmol hydrogen peroxide in 1 min is defined as 1 U of that enzyme.

The dye-mediated glucose dehydrogenase activities were measured using 0.6 mM PMS and 0.06 mM DCIP as the electron acceptors at room temperature in 10 mM potassium phosphate buffer (pH 7.0). The reduction of DCIP was measured at 600 nm. The amount of an enzyme that reduces 1 μmol DCIP is defined as 1 U, using 16.3 mM^−1^ cm^−1^ as the molar absorption coefficient of DCIP at pH 7.0.

## 4. Conclusions

We predicted the residues responsible for oxidative half reaction with oxygen, by constructing oxygen-interacting structural models of GOxs on the basis of the crystal structure of cholesterol oxidase, as well as on the fact that cholesterol oxidase and glucose oxidase enzymes are members of the GMC oxidoreductase family. Rational amino acid substitution resulted in the construction of an engineered GOx with drastically decreased oxidase activity and increased dehydrogenase activity, which was higher than that of the wild-type enzyme. As a result, the dehydrogenase/oxidase ratio of the engineered enzyme was more than 11-fold greater than that of the wild-type enzyme.

These results indicate that alteration of the dehydrogenase/oxidase activity ratio GOxs is possible by introducing a mutation into the putative functional residues responsible for oxidative half reaction with oxygen of these enzymes, resulting in a further increased dehydrogenase activity. Oxidases that are less O_2_-sensitive would be very advantageous for the development of amperometric enzyme sensors using artificial electron acceptors. So, this engineering strategy will provide an ideal enzyme for electrochemical monitoring of glucose.

## Figures and Tables

**Table 1:** The oxidase and dehydrogenase activities of wild-type GOxs and Ala-substituted mutants.

1gpe				1cf3			
	
	Ox (U/mg)	Dh (U/mg)	Dh/Ox (%)		Ox (U/mg)	Dh (U/mg)	Dh/Ox (%)
Wild-type	36 (100%)	6.5 (100%)	18.2 (100%)	Wild-type	31 (100%)	8.3 (100%)	27.1 (100%)
Ser114Ala	11 (31.2%)	25 (385%)	224 (1233%)	Thr110Ala	9.3 (30.4%)	17 (203%)	181 (668%)
Thr116Ala	n.d.	n.d.	-	Thr112Ala	1.2 (3.9%)	1.5 (17.5%)	121 (445%)
Ile219Ala	n.d.	n.d.	-				
Phe355Ala	1.1 × 10^−2^ (0.03%)	1.6 × 10^−2^ (0.24%)	145 (800%)	Phe351Ala	3.3 × 10^−2^ (0.11%)	3.6 × 10^−2^ (0.43%)	108 (339%)
Glu416Ala	n.d.	n.d.	-				
Phe418Ala	0.21 (0.58%)	0.27 (4.1%)	127 (702%)	Phe414Ala	n.d.	7.6 × 10^−2^ (0.11%)	-
Trp430Ala	0.31 (0.87%)	0.79 (12.1%)	252 (1387%)	Trp426Ala	4.4 (14.3%)	7.3 (87.3%)	165 (610%)
His563Ala	n.d.	n.d.	-				

Ox: Oxidase activity; Dh: Dehydrogenase activity; n.d.; not detected; Glucose concentration: 100 mM for 1gpe and its mutants, 200 mM for 1cf3 and its mutants.

**Table 2:** The kinetic parameters of wild-type 1gpe and Ser114Ala mutant for glucose.

	*K*_m_		*V*_max_			*V*_max_/*K*_m_	
			
	Ox (mM)	Dh (mM)	Ox (U/mg)	Dh (U/mg)	Dh/Ox	Ox (U/mg·mM)	Dh (U/mg·mM)
wild-type	8.18	9.29	227	33.3	14.6%	27.8	3.59
Ser114Ala	4.19	9.41	69.4	122	176%	16.6	13.0

Ox: Oxidase activity; Dh: Dehydrogenase activity.
